# Impact of DDT on women's health in Bangladesh: escalating breast cancer risk and disturbing menstrual cycle

**DOI:** 10.3389/fpubh.2024.1309499

**Published:** 2024-02-12

**Authors:** Zarin Subah, Jae Hyeon Ryu

**Affiliations:** College of Natural Resources and College of Agricultural and Life Sciences, University of Idaho Boise, Boise, ID, United States

**Keywords:** DDT, toxicity, breast cancer, menstruation, women's health

## Introduction

Among the array of organochlorine pesticides utilized for pest control, DDT stands as a prominent example due to its exceptional persistence and resistance to biodegradation (1–5). Comprising various derivatives such as p,p'-DDT (constituting 77 percent of DDT) and o,p'-DDT (constituting 15%), DDT finds widespread use in agricultural domains, fisheries, and vector control, notably targeting mosquitoes and other insects (6). Following the Stockholm Convention, annual averge of DDT usage was roughly 5388 metric tons from 2001 to 2007, reducing to around 3772 metric tons annually from 2008 to 2014. According to WHO, India remained the primary consumer of DDT, using it mostly to combat malaria and leishmaniasis (7). As its exposure is continuous in the environment, it is transferred to the higher consumer level of the food chain through accumulation and persistence in fatty tissues (8). DDT exhibits multifaceted effects on avian, aquatic, and, arthropod populations, precipitating species extinction, as evidenced by studies illustrating its adverse impact on birds like brown pelicans and eagles through eggshell thinning, consequently threatening habitat integrity (9, 10). Recent studies have been able to detect the impact of DDT on human beings. Recognized as a xenoestrogen, DDT disrupts the endocrine system, manifesting reproductive toxicity (11). Its proclivity for stability and bioaccumulation facilitates its entry into human adipose tissues and subsequently into breast milk. Through these lipid reservoirs, DDT infiltrates the circulatory system, affecting various physiological functions (2). Consequently, the augmented risk of cancer poses critical health threats, especially to infants consuming DDT through maternal breast milk, potentially leading to fatal consequences (10). The cumulative toxicity of DDT over prolonged exposure periods can significantly alter morphogenesis, induce carcinogenic diseases, and precipitate failures within the reproductive system (12–14).

The underlying chemical mechanism of DDT's toxicity involves its interference with the function of ATP in the shell gland membrane, notably inhibiting calcium adenosine triphosphatase (9, 15–18). DDT exerts an impact on mitochondrial metabolism in humans, potentially disrupting hepatocyte maintenance and integrity (19, 20). Mitochondrial dysfunction serves as a key contributor to chemically induced hepatic toxicity and the onset of carcinogenic disturbances (9). The metabolite p,p'-DDE [1,1'-dichloro-2,2'-bis(p-chlorophenyl)ethylene] is the primary stage of the harmful and persistent p,p'-DDT. Studies have indicated a correlation between elevated serum levels of p,p'-DDT and o,p'-DDT and an increased incidence of breast cancer in young females, particularly when DDT usage is extensive (21). This persistent toxicity poses a concerning threat to the health and wellbeing of Bangladeshi women, particularly in their role as primary caregivers and the transference of contaminants to the next generation through breastfeeding (22).

## DDT usage in Bangladesh

The historical use of DDT in Bangladesh has been mainly linked to its application in pest control, agricultural improvement, and disease control programs—most notably, the 1960s and 1970s malaria eradication efforts. The aquatic resources in Bangladesh serve as a vital component of the country's sustenance, with marine organisms and fishes being preserved and dried to meet substantial consumer demand. In the preservation process, insecticides, notably DDT, have historically been employed to deter insect-related spoilage of dried fish products (23). However, increasing concerns regarding its persistent nature in the environment, bioaccumulation in organisms, and potential adverse impacts on human health prompted regulatory interventions. Despite the official ban on DDT following the Stockholm Convention Treaty of 2007, its continued usage persists within the country (24). Research investigations have revealed varying concentrations of DDT in fishes, ranging from 3.04 to 875.0 parts per billion (ppb), with notably elevated levels observed in specific species such as ribbon fish (131.6 to 149.4 ppb), shrimp (3.04 to 318.2 ppb), and Bombay duck (61.9 to 875.0 ppb) (23, 25, 26). These findings were derived from samples collected across food markets in Dhaka and Chittagong. The absence of stringent regulatory measures in Bangladesh has resulted in the unrestricted use of several organochlorine insecticides, including DDT, without prescribed limits or guidelines (27, 28). Consequently, the lack of regulatory oversight coupled with the affordability of these insecticides has led to the potential presence of high concentrations of DDT in dried fish products, accentuating the need for comprehensive regulatory frameworks and guidance in pesticide usage (8, 29).

## Discussion: environmental and health consequences of DDT exposure

When DDT is introduced in the food chain of the environment as a pesticide, the bioaccumulation process happens where the derivatives of DDT magnify in each trophic level. DDT with its isomers enters into the human body upon inhalation or ingestion through the food web (30). These metabolites bind with the estrogenic receptors and disrupt the function and lead to carcinogenic cell development in women's bodies. The negative effect of the serum level DDT and its isomers impacting the menopausal estrogens cause an increase of breast cancer (6, 14, 31). Besides breast cancer, DDT also has an impact in disrupting menstruation cycle and creating further reproductive system failure. Polymenorrhea (occurring before the 21 days cycle) and oligomenorrhea (occurring 40 or more days apart) in ovarian function due to the impact of DDT enhances failure in pregnancy and development of cancer in the reproductive system (32, 33).

## DDT in enhancing the risks of breast cancer

In Breastfeeding, the isomer of DDT which is p,p'-DDT is transferred into breast milk from its usage for the interruption of malaria spreading (34). Studies show that malaria affected areas where DDT is used to prevent mosquito growth and it has been found that a high amount of DDT exists in breast milk (35–38). Based on the assessment of the literature, it is possible that Bangladeshi women's breast milk contains significant concentrations of DDT, particularly in the regions of the country where malaria is endemic and DDT is used to control mosquitoes (39). However, there are no specific research in Bangladesh that investigate this correlation directly. Furthermore, DDT is found to be conveyed to infants as their blood serum is found contaminated with DDT (1, 33).

The *in utero* exposure of DDT enhances the risk of breast cancer through several deleterious impacts in breast and genital tract which leads to the development of carcinogenic cells (14, 40). The malformation in this process leads to cancer and it has probability to affect not only the mother but also the off-springs of the mother. The high level of o,p'-DDT in the serum of mother can result in future risk of breast cancer in her daughter. The process happens in a way where the receptor of estrogen and progesterone becomes positive and the protein kinase erbB-2, a receptor tyrosine (also called HER2) becomes negative. This process can result in vital effects in the women who are below 14 years old. The risk increases 5-fold for breast cancer for them and also further transferred in daughters breastfed by those mothers. Thus, the researchers assure that the risk is increased in women who are exposed to DDT during mammogenesis, which means the period between fetal and pubertal development. The remodeling of carcinogenic tissues happens in adulthood. DDT acts like an environmental endocrine disruptor altering the endogenous hormones and breast morphogenesis (4).

## Relationship of DDT and menstruation cycle disruption

Exposure of p,p'-DDT through several pesticides ingested by women from several food items create disruption in hormone signaling (41). The polychlorinated biphenyls (PCBs), from p,p'-DDTs affect the function of the hypothalamus-pituitary-ovarian axis besides hormone signaling. The chemical reaction among these shortens the menstruation cycle and affects the reproductive system of the creation of ovum (42, 43). o,p'-DDT which is the isomer of DDT and its metabolic products such as DDE and DDD with their isomers o,p'-DDD, o,p' DDE have binding assay with women's estrogen receptor. Besides, the other two isomers of DDT, such as p,p'-DDT and also p,p'-DDE have high affinity to bind with progesterone receptors which play a role in maintaining menstruation cycle and pregnancy (44). These binding affinities of DDT severely affect the endocrine function and lead to reproductive system failure in women. A study has found that the luteal phase in the menstrual cycle decreases with 0.6 days with the doubling rate (2 × ) of DDT isomer p,p'-DDE and for quartile increase (4 × ) of it decreases the metabolic activities of progesterone (2).

## Effect of DDT exposure detected in Bangladesh women health

Bangladesh, being predominantly agrarian, has witnessed a surge in the usage of persistent organochlorine insecticides like DDT, HCH, PCB, and HCB in recent years. These compounds, characterized by their enduring nature and limited biodegradability, persist within the environment over extended periods (45). Despite the prohibition of DDT in Bangladesh, its continued use in agriculture, fisheries, and vector control has resulted in elevated concentrations detected notably in food sources such as fish (25, 46). Consequently, high levels of DDT have been identified in the plasma cells of Bangladeshi individuals due to the ingestion of DDT-contaminated foods (47).

The impact of DDT on Bangladeshi women, particularly those of reproductive age, demonstrated complex relationships between dietary patterns and increased exposure to this substance, most notably through food consumption and a variety of exposure routes (23). Studies have indicated a substantial disparity in the concentration of organochlorine insecticides, such as DDT, in Bangladeshi mothers' breast milk compared to levels observed in Europe and the United States (48). This significant variance suggests an augmented risk for mothers incurring breast cancer and raises concerns regarding potential implications for daughters who are breastfed or exposed to organochlorines like DDT transmitted through maternal sources. Breast cancer affects ~13,000 women in Bangladesh each year, and over 7,000 of them die as a result of the disease. The incidence of breast cancer in the country is expected to be 22.5 per 100,000 females of any age and it is the most common among women aged 15 to 44 years old (49, 50). Also, a significant correlation is found between BMI, education level, and serum p,p'-DDE concentration, suggesting that high socio-economic profile women tended to consume expensive, higher-fat foods like meat and fatty fish, influencing increased DDT exposure (22–24). Moreover, a weak positive link between p,p'-DDE levels and beef consumption among Nullipara women hinted at beef and fish being significant contributors to DDT intake in Bangladesh (24). Beef emerged as a key route of DDT intake for reproductive-age women in Bangladesh. DDT concentrations were notably high in meat, house dust, and breast milk, indicating varied exposure sources. While daily DDT intake levels didn't surpass WHO guidelines, p,p'-DDT levels exceeded the US EPA's oral reference dose. Another study in the Dhaka city, Bangladesh, detected substantial p,p'-DDT levels in cord blood of pregnant mother-newborn in Bangladesh, signifying recent DDT exposure (22, 51). Nevertheless, the future risk of the mother suffering from breast cancer and also the daughter who is breastfed or consuming organochlorine i.e., DDT by those mothers can also be affected by reproductive system failure or breast cancer (52–56).

## Conclusion

The reason behind the continued usage of DDT in Bangladesh is the lack of consciousness among people about its adverse effect and there is strict regulation in prohibiting its usage. The fatal effect of DDT needs to be researched more in Bangladeshi mothers and their daughters inheriting DDT based carcinoma living in different cities of Bangladesh. Besides this, the harmful impacts DDT in human health needs to be conveyed in farmers and fishermen who continuously use DDT so that consciousness can be built to stop the usage of DDT. Also, the alternative bio-pesticides which are easily biodegradable need to be introduced to Bangladeshi people in order to reduce the use of DDT and encourage the use of eco-friendly components that benefit both the environment and human health.

## Author contributions

ZS investigated the proposed research and wrote the manuscript. JR provided supervised directions as an academic advisor at the University of Idaho.
